# Characterization of the Complete Mitochondrial Genome of *Schizothorax kozlovi* (Cypriniformes, Cyprinidae, Schizothorax) and Insights into the Phylogenetic Relationships of *Schizothorax*

**DOI:** 10.3390/ani14050721

**Published:** 2024-02-25

**Authors:** Qiang Qin, Lin Chen, Fubin Zhang, Jianghaoyue Xu, Yu Zeng

**Affiliations:** 1 College of Environmental Science and Engineering, China West Normal University, Nanchong 637009, China; sczhangfubin@163.com (F.Z.); xujianghaoyue@163.com (J.X.); 2 Powerchina Chengdu Engineering Corporation Limited, Chengdu 611130, China; 18780044276@163.com; 3 College of Life Science, China West Normal University, Nanchong 637009, China

**Keywords:** *Schizothorax kozlovi*, *Schizothorax*, mitochondrial genome, phylogenetic analysis, fish genetics

## Abstract

**Simple Summary:**

As an endemic and vulnerable fish from the upper Yangtze River in China, *Schizothorax kozlovi* holds significant scientific and ecological importance, yet it has received little attention so far. In this paper, we reported the characterization of the mitochondrial genome of *S. kozlovi*, and further investigated the phylogenetic relationships of *Schizothorax*. The results showed that the mitochondrial genome of *S. kozlovi* had a total size of 16,585 bp, a circular arrangement, and contained 13 PCGs, 22 tRNAs, two rRNAs, and two non-coding regions. Moreover, the phylogenetic analyses demonstrated that *Schizothorax* could be classified into four clades, and *S. kozlovi* was closely related to *Schizothorax chongi*. The present study enriched the basic biological data for *S. kozlovi* and provided fundamental references for the conservation of *S. kozlovi* and *Schizothorax*.

**Abstract:**

*Schizothorax kozlovi* is an endemic and vulnerable fish species found in the upper Yangtze River in China. Over the past few years, the population resources of *S. kozlovi* have been nearly completely depleted owing to multiple contributing threats. While the complete mitochondrial genomes serve as important molecular markers for phylogenetic and genetic studies, the mitochondrial genome of *S. kozlovi* has still received little attention. In this study, we analyzed the characterization of the mitochondrial genome of *S. kozlovi* and investigated the phylogenetic relationships of *Schizothorax*. The complete mitochondrial genome of *S. kozlovi* was 16,585 bp in length, which contained thirty-seven genes (thirteen protein-coding genes (PCGs), two ribosomal RNA genes (rRNAs), twenty-two transfer RNA genes (tRNAs)) and two non-coding regions for the origin of light strand (OL) and the control region (CR). There were nine overlapping regions and seventeen intergenic spacers regions in the mitochondrial genome. The genome also showed a bias towards A + T content (55.01%) and had a positive AT-skew (0.08) and a negative GC-skew (−0.20). All the PCGs employed the ATG or GTG as the start codon and TAA, TAG, or single T as the stop codon. Additionally, all of the tRNAs displayed a typical cloverleaf secondary structure, except trnS1 which lacked the D arm. The phylogenetic analysis, based on the maximum likelihood (ML) and Bayesian inference (BI) methods, revealed that the topologies of the phylogenetic tree divided the *Schizothorax* into four clades and did not support the classification of *Schizothorax* based on morphology. The phylogenetic status of *S. kozlovi* was closely related to that of *S. chongi*. The present study provides valuable genomic information for *S. kozlovi* and new insights in phylogenetic relationships of *Schizothorax*. These data could also offer fundamental references and guidelines for the management and conservation of *S. kozlovi* and other species of *Schizothorax*.

## 1. Introduction

The mitochondria, as fundamental organelles, are widely found in eukaryotic cells and are associated with pivotal roles such as energy metabolism, genetic signaling, biological synthesis, and cell apoptosis [1]. Since the discovery of the mitochondria, researchers have never ceased to explore their structure and function. Particularly with the advancement of molecular biotechnology in recent years, the mitochondrial genome has received a great deal of attention. Several studies have shown that the mitochondria of animals contain an independent genome and that the mitochondrial genome is a closed-circular DNA molecule ranging in size from 14 kb to 20 kb with a high degree of autonomy in terms of genetic replication, transmission, and expression [2]. The mitochondrial genome typically consists of thirty-seven genes, including thirteen protein-coding genes (PCGs), twenty-two transfer RNA genes (tRNAs), two ribosomal RNA genes (12S rRNA and 16S rRNA), and two non-coding regions (origin of the light strand (OL) and the control region (CR)), except for a few species (e.g., *Crassostrea gigas*, *Metridium senile*) with variations in the characterization of the genome [3]. Moreover, mitochondrial genome sequencing analysis revealed that the mitochondrial DNA (mtDNA) is a double-stranded molecule with the heavy chain on the outer ring and the light chain on the inner ring, but the majority of genes are transcribed by the heavy chain. The rapid advances in sequencing technology over the past few years have facilitated the availability of the mtDNA, which has led to a boom in the field of animal mitochondrial genome research, particularly in vertebrates such as fishes, amphibians, reptiles, and birds [4,5,6,7]. As the study progressed, researchers have summarized that mtDNA is characterized by small size, simple structure, maternal inheritance, missing intron, high evolutionary rate, conserved content, and easy amplification [8,9,10]. Based on the advantages of genomic characterization above, the mtDNA has been employed as a useful molecular marker in the study of molecular evolution, biodiversity analysis, phylogenetic reconstruction, population genetic assessment, and genomic comparison [11,12]. In the past decade, there also has been a flourish in the sequencing of fish mitochondrial genomes. For example, analyses of gene localizations, tRNA secondary structures, and the transcription and replication regions within the mitochondrial genome have provided a wealth of reliable information on fish taxonomic classification, ecological adaptation, and phylogenetic relationships, reminding us that the complete mitochondrial genome are important molecular resources for the management and conservation of fishes [13].

*Schizothorax kozlovi* Nikolsky, 1903 belongs to Cypriniformes, Cyprinidae, *Schizothorax* and is mainly distributed in the Jinsha River, Yalong River, and Wujiang River basins [14]. In addition, *S. kozlovi* is considered to be a typical plateau cold-water fish and it is also an endemic and economic fish found in the upper Yangtze River in China. The *S. kozlovi* has been an important fish with great value in ecology, the economy, and scientific research. Unfortunately, recent field surveys have revealed that population resources of *S. kozlovi* were significantly decreased and close to being depleted due to overfishing, water pollution, climate change, and habitat fragmentation [15]. *S. kozlovi* has also been recognized as a vulnerable species (VU) in the IUCN Red List [16]. Therefore, research on and attention to the biology of *S. kozlovi* are urgently needed for sustainable resource conservation. Previously, researchers have conducted studies on growth, reproduction, diet, population, and diversity [15,17,18,19,20,21]. But there has been little focus on the characterization of the mitochondrial genome and phylogenetic analysis for *S. kozlovi*. Only a few sequences of mitochondrial genome are included in the GenBank database (e.g., NC 027670.1) and Wang et al. [22] incompletely explored the phylogenetic relationships of some fish species in the *Schizothorax*. Morphologically, the *S. kozlovi* had some unique features such as a long and flat body covered in tiny scales, an inconspicuous keratin at the anterior edge of the jaw, a strong dorsal fin, two pairs of beards, and several pairs of developed anal scales [14,23]. These traits provided the basis for behaviors associated with adaptation to the low temperature and hypoxia of the plateau habitats [24,25]. In addition, the mitochondrial genome which encodes its functional genes might provide some key evidence about its adaptability, yet the complete mitochondrial genome data of *S. kozlovi* have rarely been reported and analyzed.

Over the past century, the fish species *Schizothorax* has received great interest from researchers. The Schizothorax is widely distributed across the Tibetan Plateau and is a representative example of the adaptation of native fish species to the plateau habitat. Several studies have suggested that the evolution process of *Schizothorax* is closely related to the uplift process in the Tibetan Plateau [26,27,28]. Specifically, Chen and Cao [29] summarized that the *Schizothorax* is the largest genus for the subfamily of Schizothoracinae living in China. Hence, *Schizothorax* is an excellent model to explore the process of fish species’ evolution and differentiation in a complex habitat. However, the controversies over the taxonomic and phylogenetic relationships of the *Schizothorax* have long existed because of their extreme morphological and ecological similarity. For example, Heckel [30] recommend that the *Schizothorax* can be divided into three groups based on the shape of the head and the morphology of the mouth; Chen et al. [31] suggested that the *Schizothorax* should be classified into two subgenera on the basis of the presence or absence of keratin at the anterior edge of the jaw. Moreover, researchers have previously used single mitochondrial gene such as Cyt b or COI to determine phylogenetic relationships for species among *Schizothorax*, which may lead to one-sided and biased conclusions [21,22,32]. Within this context, it is necessary to unite multiple makers from genomes to investigate the taxonomic and phylogenetic relationships for *Schizothorax*.

In this study, we reported the complete mitochondrial genome sequence of *S. kozlovi*; described the characterization of mitochondrial genome such as genome composition, organization, annotation; and reconstructed a phylogenetic tree based on the sequences within the mitochondrial genome. From this, we aimed to comprehensively understand the complete mitochondrial genome of *S. kozlovi*, to determine the potential phylogenetic status of *S. kozlovi*, and to explore the phylogenetic relationships of *Schizothorax*. Therefore, the results will provide fundamental molecular data for resources management and the conservation of *S. kozlovi* and help us form a better understanding of the phylogenetic relationships of *Schizothorax*.

## 2. Materials and Methods

### 2.1. Sample Collection and DNA Extraction

The specimen of *S. kozlovi* was collected from the Zangqu River (31°34′ N, 98°24′ E), a tributary of the upper Jinsha River, China. Once captured, the specimen was identified to species level according to the main ideas about morphology referring to Ding [14], Chen [31], and Wu [23]. Then, 2–3 g of muscle tissue was cut from the left side of the back and tagged with a label for the specimen. Subsequently, the specimen and muscle tissue were immediately preserved in 95% ethanol and kept in a refrigerator at −20 °C until taken back to the laboratory. Finally, the specimen and muscle tissue were stored in the College of Life Science at China West Normal University until DNA extraction. The experiments on *S. kozlovi* in this study were conducted in accordance with the national guidelines for laboratory animal care and treatment. This study was approved by the Institutional Review Board of China West Normal University.

The total genomic DNA from left back muscle tissue was extracted by using E.Z.N.A.^®^ Tissue DNA Kit (Origin gene Co. Ltd., Shanghai, China) in accordance with the manufacturer’s guidelines. The genomic DNA was analyzed with 1% agarose gel electrophoresis to detect the DNA quality. And the extracted DNA was stored at −20 °C for subsequent use.

### 2.2. Genome Sequencing and Assembly

In this study, next-generation sequencing (NGS) was employed to obtain the complete mitochondrial genome sequence of *S. kozlovi*. Firstly, the genomic DNA was fragmented into 300–500 bp size fragments by Covaris M220, target fragments were amplified by polymerase chain reaction (PCR), and the library was constructed by TruseqTM RNA sample Prep Kit (Illumina, San Diego, CA, USA) with 1 µg DNA. Then, the mitochondrial genome sequence was performed on Illumina NovaSeq6000 platform at Origin gene Co. Ltd., Shanghai, China. Raw reads were converted from Illumina sequencing into FASTQ format by FastQC v0.11.4 [33]. Moreover, the raw data were cleaned up so that the low quality reads (<Q20), high “N” ratio sequences (>10%), fragments with length less than 50 bp were filtered out to obtain high quality clean data for quality control [34]. And multiple iterations of splicing for clean data were performed by Getorganelle v1.7.0 [35] and corrected by using Pilon v1.23 [36]. Finally, the clean data were assembled with the de novo assembly program to obtain complete mitochondrial genome sequence by NOVOPlasty v4.2 [37].

### 2.3. Sequence Annotation and Analysis

The mitochondrial genome of PCGs, rRNAs, and tRNAs were annotated in the MITOS2 webserver (http://mitos2.bioinf.uni-leipzig.de/index.py, accessed on 23 December 2021) [38]. The secondary structures of tRNAs from *S. kozlovi* were initially predicted by the tRNAscan webserver (http://lowelab.ucsc.edu/tRNAscan-SE/, accessed on 18 May 2023) [39]. The circular mitochondrial genome map was generated by OGDRAW v1.2 [40]. The relative synonymous codon usage (RSCU) for protein-coding genes, start/stop codon, and nucleotide composition were analyzed in the MEGA v7.0 [41] and the Codonw v1.4.4 (https://sourceforge.net/projects/codonw/, accessed on 23 December 2021). The skew values of the nucleotide composition was measured based on the following formulas: AT-skew = (A − T)/(A + T) and GC-skew = (G − C)/(G + C), where a positive AT-skew/GC-skew value represents that there are more As than Ts; while a negative AT-skew/GC-skew value means that there are less As than Ts [42,43]. The complete mitochondrial genome sequence of *S. kozlovi* was submitted to the GenBank database with accession number OR416862.1.

### 2.4. Phylogenetic Analysis

To clarify the phylogenetic status of *S. kozlovi* and understand the phylogenetic relationships within *Schizothorax*, available mitochondrial genome sequences for 32 species of *Schizothorax* and two species of *Spinibarbus denticulatus* and *Spinibarbus sinensis* from *Spinibarbus* as outgroup were downloaded from the GenBank database (https://www.ncbi.nlm.nih.gov/, accessed on 10 January 2024) (Table 1). The nucleotide sequences of thirteen PCGs, twenty-two tRNAs, and two rRNAs from mitochondrial genome for all species (a total of 35 species including *S. kozlovi*) were employed to reconstruct the phylogenetic tree. In this study, all procedures, including sequence extraction, alignment, and concatenation were performed in Phylosuite v1.2.1, which integrated multiple software programs for phylogenetic analyses [44]. After that, based on the concatenated sequence matrix, Bayesian inference (BI) and maximum likelihood (ML) methods were applied to determine phylogenetic trees. The selection of the best-fit substitution model for phylogenetic analyses was carried out by ModelFinder integrated in Phylosuite [45]. The maximum likelihood (ML) analysis was inferred by IQ-TREE integrated in Phylosuite, which applied the automatically selected option of the model in IQ-TREE for 5000 ultrafast bootstrap replicates [46,47]. The Bayesian inference (BI) analysis was deduced by MrBayes integrated in Phylosuite with four simultaneous Markov chain Monte Carlo (MCMC) algorithms [48]. Two independent runs of 1,000,000 generations were performed for sampling every 1000 generations, in which the initial 25% data were discarded as burn in and BI analysis was considered to be achieved since the average standard deviation of split frequencies was below 0.01 [49]. The final phylogenetic trees were viewed in iTOL v6.8.1 (https://itol.embl.de/, accessed on 2 February 2024).

## 3. Results

### 3.1. Genome Organization and Composition

**Figure 1:**
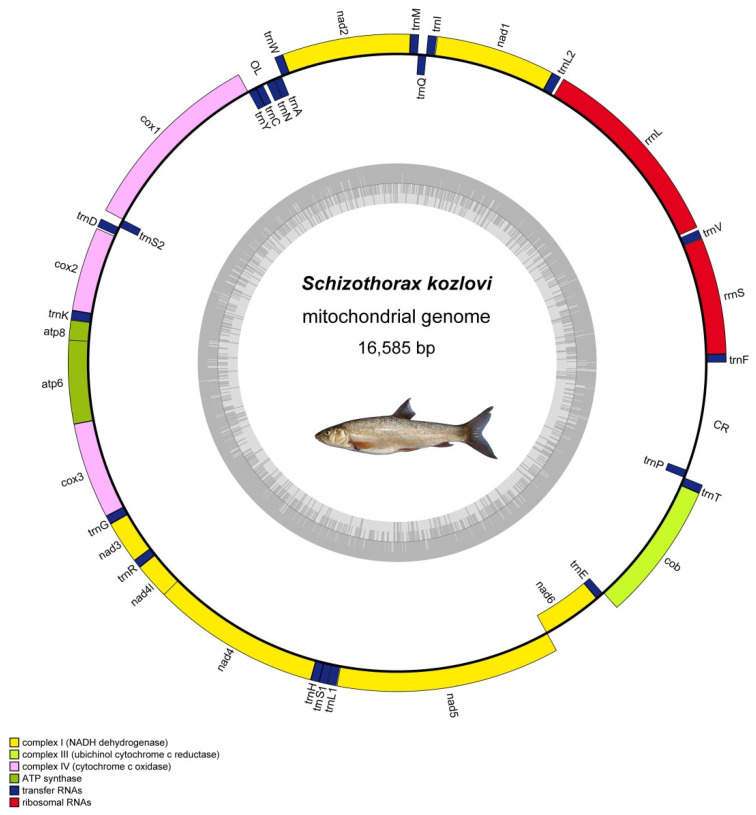
A circular map of the mitochondrial genome of *S. kozlovi*. The inner ring is light strand and the outer ring is heavy strand. The thirteen PCGs, two rRNAs, twenty-two tRNAs, and two non-coding regions of OL and CR are shown on the circular map of mitochondrial genome. The blue strips represent transfer RNA genes; the red strips represent ribosomal RNA genes; the yellow strips, light green strips, pink strips, dark green strips represent protein-coding genes for NADH dehydrogenase, cytochrome c oxidase, cytochrome b, ATP synthase, respectively.

The complete mitochondrial genome of *S. kozlovi* is a closed, circular molecule with a total length of 16,585 bp (Figure 1). And the obtained mitochondrial genome contained thirty-seven genes, including thirteen protein-coding genes (PCGs), two ribosomal RNA genes (rRNAs), twenty-two transfer RNA genes (tRNAs), and two non-coding regions for the origin of light strand (OL) and control region (CR) (Table 2). Among these genes, nine genes with the one protein-coding gene nad6 and eight transfer RNA genes, trnQ, trnA, trnN, trnC, trnY, trnS2, trnE, trnP, were encoded on the light strand (L), while twenty-eight genes including twelve protein-coding genes, fourteen transfer RNA genes, and two ribosomal RNA genes were located on the heavy strand (H) (Figure 1). Specifically, there were nine overlapping regions in the mitochondrial genome of *S. kozlovi*, which comprised a total length of 29 bp and varied in size from 1 to 7 bp, and the longest overlapping regions were located between the atp8 and atp6 and nad4l and nad4 genes (Table 2). In addition, 17 intergenic spacers regions with a total length of 201 bp were found in the mitochondrial genome, which ranged in size from 1 to 102 bp and the largest intergenic spacer region lied between CR and trnF genes (Table 2).

The nucleotide compositions of the complete mitochondrial genome for *S. kozlovi* were 29.59% for A, 25.42% for T, 17.94% for G, and 27.05% for C (Table 3). The nucleotide composition analysis also showed that the A + T content (55.01%) was greater than the G + C content (44.99%) for the mitochondrial genome and the A + T contents in different genes such as PCGs (54.27%), tRNAs (54.80%), rRNAs (54.70%), and CR (66.87%) were higher than G + C content, indicating a bias towards A + T content in the nucleotide composition of *S. kozlovi* (Table 3). Additionally, the AT-skew had a positive value (AT-skew = 0.08) and the GC-skew had a negative value (GC-skew = −0.20) for the complete mitochondrial genome, which implied that A had a higher abundance than T and C had a higher occurrence than G (Table 3).

### 3.2. Protein-Coding Genes and Codon Usage

The results of this study showed that the complete mitochondrial genome of *S. kozlovi* had 13 PCGs with a total length of 11,415 bp accounting for 68.83% of the mitochondrial genome (Table 3). The PCGs consisted of seven NADH dehydrogenases (nad1, nad2, nad3, nad4, nad4l, nad5, nad6), three cytochrome c oxidases (cox1, cox2, cox3), two ATP synthases (atp6 and atp8), and one cytochrome b (cob) (Figure 1). Moreover, 12 PCGs were encoded on the light strand (L), while only nad6 was encoded on the heavy strand (H). The nucleotide compositions of the PCGs were as follows: A = 27.03%, T = 27.24%, G = 17.88%, and C = 27.85%. Both the AT-skew and GC-skew values of the PCGs were found to be negative: AT-skew = −0.003 and GC-skew = −0.22, indicating that there was more T and C in the majority of PCGs (Table 3).

**Figure 2:**
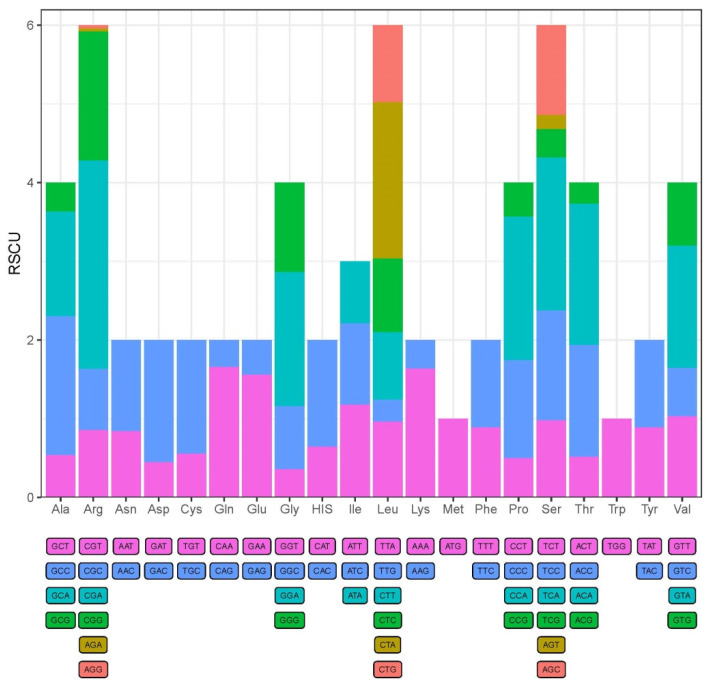
The relative synonymous codon usage (RSCU) for PCGs in the complete mitochondrial genome of *S. kozlovi*. The different colors represent different codon families corresponding to amino acids.

The 13 PCGs of the mitochondrial genome totally encoded 3653 amino acids. All the PCGs used the ATG or GTG as the start codon, where 12 of the 13 PCGs had a start codon of ATG and only the codon for cox1 started with GTG (Table 2). The stop codons were represented by TAA, TAG or single T, where three PCGs (cox2, nad4, cob) were terminated by the stop codon of single T, three PCGs (nad2, nad3, atp8) employed TAG as a stop codon, and the remaining six PCGs (nad1, nad4l, nad5, nad6, cox1, cox3, atp6) were terminated by the TAA stop codon (Table 2). The relative synonymous codon usage (RSCU) values for the 13 PCGs are visualized in Figure 2. The results of RSCU for *S. kozlovi* suggested that the most commonly used codons were CGA (Arg), CTA (Leu), TCA (Ser), CCA (Pro), ACA (Thr), and we can also conclude that the codon usage of PCGs was biased toward amino acids encoded by A-rich and C-rich codons.

### 3.3. Transfer RNAs, Ribosomal RNAs, and Noncoding Regions

**Figure 3:**
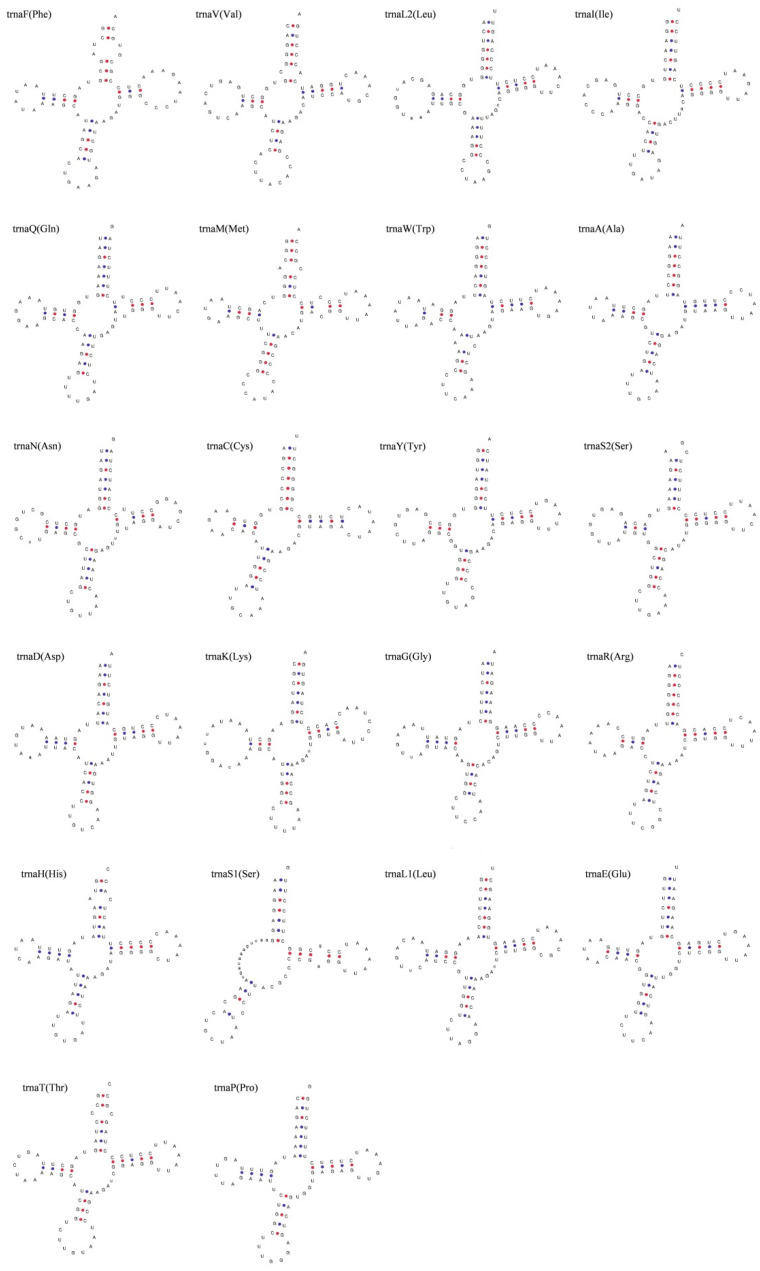
The inferred secondary structures of tRNAs in the complete mitochondrial genome of *S. kozlovi*. The 22 tRNA genes were labeled with standard abbreviations in the top left and the corresponding amino acids for translocation were listed in parenthesis, respectively.

The complete mitochondrial genome of *S. kozlovi* contained twenty-two transfer RNAs (tRNAs), two ribosomal RNAs (rRNAs), and two non-coding regions (OL and CR). The tRNAs ranged in size from 67 to 76 bp with a total length of 1562 bp (Table 2). It is also clear that eight tRNAs were located on the light strand (L), while the other fourteen tRNAs were encoded on the heavy strand (H). The tRNAs also had an A + T bias so that the A + T content (54.80%) was higher than the G + C content (45.20%) (Table 3). And the tRNAs showed positive values for AT-skew (0.03) and GC-skew (0.05), indicating that the tRNAs were slightly biased towards A and G (Table 3). The inferred secondary structures of the tRNAs supported that 21 of the 22 tRNAs can be folded into the typical cloverleaf secondary structure, which is made up of four domains (AA stem, D arm, AC arm, and T arm), but the other tRNAs of trnaS1 (Ser) was found to lack the D arm in its secondary structure (Figure 3). Additionally, the results also showed that the typical G-C and A-U base pairs were presented in all of the tRNAs. But some mismatched base pairs were also identified in the base pairs, for example 15 tRNAs including trnF, trnL2, trnM, trnW, trnA, trnC, trnY, trnS2, trnD, trnK, trnG, trnH, trnS1, trnE, trnP had G-U or U-G base pairs, and the U-U base pairs appeared in trnF, trnQ, and trnN (Figure 3).

The two rRNAs of rrnS and rrnL were both encoded on the heavy strand (H) with lengths of 954 bp and 1629 bp, respectively (Table 2). The rrnS and rrnL were located between trnF and trnL2 and the trnV was interspersed between rRNAs. The rRNAs also had an A + T bias meaning that the A + T content was 54.70%. The AT-skew value for the rRNAs was positive (AT-skew = 0.26), while the GC-skew value for the rRNAs was slightly negative (GC-skew = −0.06) (Table 3). The non-coding regions of the mitochondrial genome of *S. kozlovi* mainly included the origin of the light strand (OL) and the control region (CR). The OL was located between trnN and trnC with a length of 32 bp. The CR was a relatively long non-coding region with a length of 821 bp, which was located between trnP and trnF and there was an intergenic spacer of 102 bp between the CR and trnF (Figure 1; Table 2). The OL had a significant G + C bias with a G + C content of 65.62%, while the CR had a significant A + T bias with an A + T content of 66.87% (Table 3). The AT-skew values and GC-skew values for OL and CR were negative, indicating that there was more T and C for OL and CR.

### 3.4. Phylogenetic Relationships

**Figure 4:**
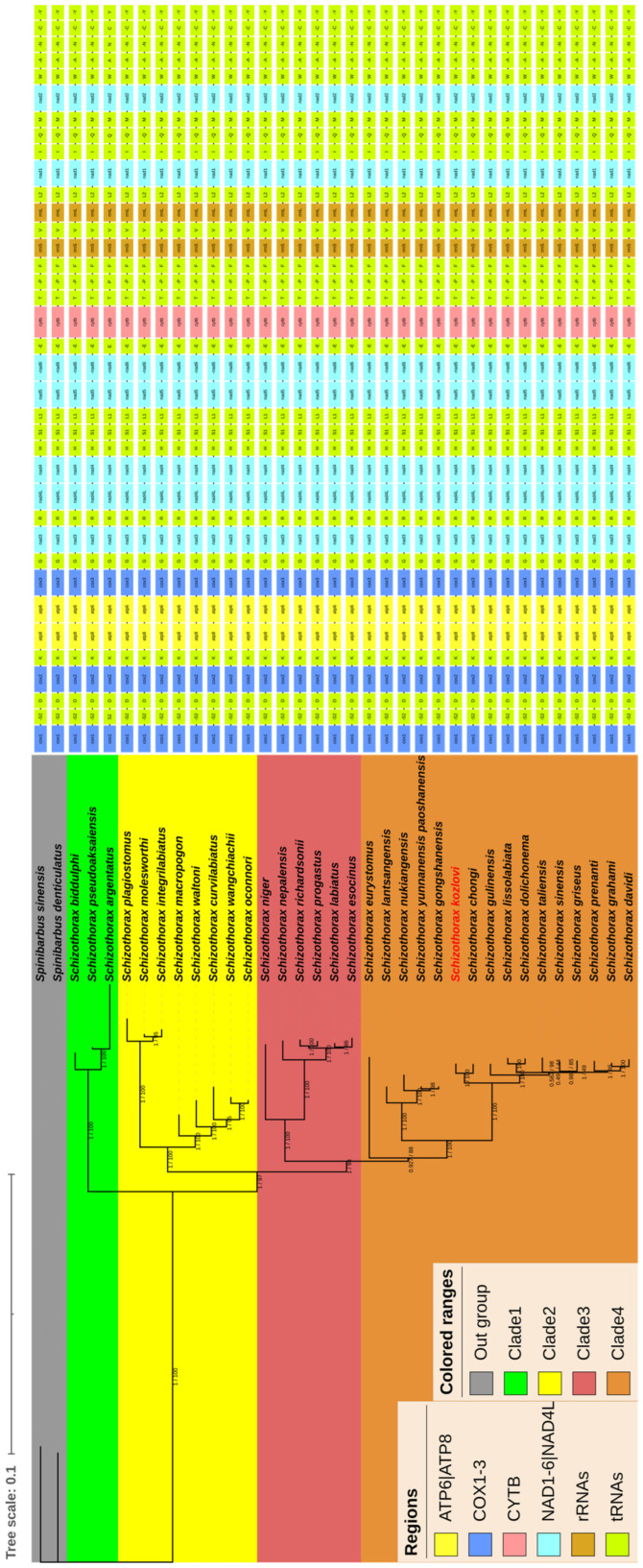
The phylogenetic tree reconstructed from the nucleotide sequences of thirteen PCGs, twenty-two tRNAs, and two rRNAs using BI and ML methods. Numbers on branches represent posterior probabilities (BI) and bootstrap percentages (ML), respectively. Different colors in regions represent different genes, and gene orders are listed behind the phylogenetic tree. Different colors in the phylogenetic tree represent outgroups and clades. The species of *S. kozlovi* determined in the present study is marked in red.

In this study, we determined the phylogenetic relationships of thirty-three species of *Schizothorax* with two species of *Spinibarbus* according to the concatenated nucleotide sequences from thirteen PCGs, twenty-two tRNAs, and two rRNAs from mitochondrial genomes based on ML and BI analyses. As a result, the phylogenetic trees generated by the ML and BI methods had the same topological structure and showed well-supported branches. Thus, the current study only used the phylogenetic tree generated through the ML analysis including posterior probabilities and bootstrap values, as shown in Figure 4. The results of the phylogenetic tree showed that the majority of nodes had high support rates with bootstrap proportions ≥ 85 and posterior probabilities ≥ 0.927. While a few nodes in the branches had relatively low support rates, for instance, nodes in the branches had the lowest support rates with a bootstrap proportion of 44 and a posterior probability of 0.49. The results of phylogenetic tree also suggested that the *Schizothorax* could be classified into four clades: Clade 1 consisted of three species, Clade 2 consisted of eight species, Clade 3 consisted of six species, and Clade 4 consisted of sixteen species (Figure 4). In addition, the topologies of the phylogenetic tree exhibited that the *S. kozlovi* belonged to Clade 4 and had a relatively close phylogenetic relationship with *S. chongi*. Thus, the *S. kozlovi* and *S. chongi* formed a sister group, which together formed sister groups to other species such as *Schizothorax gulinensis*, *Schizothorax dolichonema,* and *Schizothorax lissolabiata* in Clade 4 (Figure 4).

## 4. Discussion

In this study, the characterization of the complete mitochondrial genome of *S. kozlovi* was described for the first time. Our results indicated that the mitochondrial genome of *S. kozlovi* is a closed circular structure with a total size of 16,585 bp, which is similar to other reported species of *Schizothorax* such as *Schizothorax davidi*, *Schizothorax argentatus,* and *Schizothorax prenanti*. Wang et al. [50] suggested that the size of the mitochondrial genome length in closely related species may be caused by variations in the tandem repeat elements within the CR which can explain the gene overlaps. In addition, we also found that the mitochondrial genome of *S. kozlovi* was composed of thirty-seven genes (thirteen PCGs, twenty-two tRNAs, two rRNAs) and an OL and a CR; the genome organization and composition of which were in accordance with those of most of fish species reported previously [51,52,53]. The AT and GC skews are a measure of asymmetry in the nucleotide composition of the mitochondrial genome. In this study, the A + T content was higher than the G + C content; A had a higher abundance than T so AT-skew was 0.08, and C had a higher occurrence than G so GC-skew was −0.20. Numerous studies have shown that almost all postnatal animals including fishes are characterized by AT bias, indicating that the AT-rich regions might represent the origin of replication and be more easily changed in evolution [54,55]. Generally, the strand skew biases were found to have a negative AT skew and positive GC skew. But, we summarized that the variation in AT and GC skews for different species may change slightly, which can be used as reference evidence for assessing the phylogenetic status and relationships of identified species.

Moreover, it was found that the start codons for 13 PCGs were ATG or GTG, which is consistent with the fish species of *Schizothorax wangchiachii* and *Schizothorax macropogon* in *Schizothorax* [56,57]. In this study, the majority of PCGs were represented by complete stop codons of TAA or TAG. Specifically, three PCGs (cox2, nad4, cob) exhibited incomplete stop codons of single T. Zhang et al. [58] reported that the incomplete stop codons in some PCGs were a common feature of most species of vertebrates. But we also found that there are differences in the incomplete stop codons between species from distinct taxa. For example, Mar-Silva [51] analyzed the mitochondrial genome of *Ophisternon infernale* and found that seven PCGs had incomplete stop codons for atp6, nad5 used TA as a stop codon, and cox2, cox3, nad4, nad5, and cob employed a single T as the stop codon. However, it is assumed that the incomplete stop codons of TA or T can be modified to TAA via post-transcriptional polyadenylation [59]. The relatively synonymous codon usage (RSCU) can represent the characteristics of codon usage bias in the mitochondrial genome. The results of RSCU analysis for PCGs of *S. kozlovi* in the current study showed that the most frequently used codons were CGA, CTA, TCA, CCA, and ACA, which encoded the amino acids for Arg, Leu, Ser, Pro, and Thr, respectively. Previous studies have shown that the codon usage relating to functional gene expression and protein sequence encoding might be correlated with natural selection for species [60]. The *S. kozlovi* is a typical fish species adapted to plateau habitats, thus the codon usage analysis of PCGs in mitochondrial genome can provide an important basis for understanding the origin and evolution of *Schizothorax*.

The results also showed that, with the exception of trnS1 (Ser) which lacked the D arm, all of the other tRNAs exhibited a canonical cloverleaf secondary structure. Generally, the secondary structure is essential for tRNAs to be associated with stability and function, and changing or removing key parts of the secondary structure of tRNAs might potentially affect amino acid recognition and protein biosynthesis [61]. Yet several studies have reported that trnS1 from most species of vertebrates lacked a D arm and the effects of the loss of D arm on function could be compensated for by other interactions [62,63]. Additionally, our study indicated that the stems of secondary cloverleafs for tRNAs included mostly normal base pairs and some mismatched base pairs. Specifically, trnF, trnL2, trnM, trnW, trnA, trnC, trnY, trnS2, trnD, trnK, trnG, trnH, trnS1, trnE, trnP had G-U or U-G base pairs, and trnF, trnQ, trnN had U-U base pairs. According to Varani and McClain [64], the G-U and U-U wobble base pair is the most common non-Watson–Crick base pair, and the G-U or U-U base pairs have a thermodynamic stability comparable to that of Watson–Crick base pairs and are nearly isomorphic to them, so they would be likely to be the substitutions for a G-C or A-U base pair. The non-coding regions of the mitochondrial genome of *S. kozlovi* mainly included the origin of the light strand (OL) and the control region (CR), which were closely related to the initiation of the replication and transcription of the mitochondrial genome. Similarly to other fish species, the OL had a relatively short length and usually folded into a hairpin secondary structure [65,66]. The CR in the mitochondrial genome of *S. kozlovi* had the highest A + T content (66.87%), indicating that the sequence contained AT-rich regions. Previous studies have shown that the CR is mostly a tandem repeat sequence and determines the replication and transcription of the mitochondrial genome. The CR is also considered to be under less selective pressure, and it can, therefore, replicate more efficiently and keep the most rapid evolutionary rate [67,68]. Hence, we can infer that the tandem repeat sequence of different copies as well as the insertion or deletion of gene fragments in the CR might be responsible for variations in the molecular size of the mitochondrial genome of *Schizothorax*.

In this study, we reconstructed a phylogenetic tree for *Schizothorax* based on mitochondrial genomes. The results provided some insights into the phylogenetic status of *S. kozlovi* and its phylogenetic relationships with of *Schizothorax*. Previously, Rustam et al. [56] conducted a study on the phylogenetic relationships of Schizothoracinae based on 13 PCGs from the mitochondrial genome, and classified *Schizothorax* as a primitive taxon. Wang et al. [22] inferred the phylogenetic relationships using the 13 PCGs for 28 species and found that 13 species of *Schizothorax* were clustered into a clade. However, these studies did not provide specific analyses of the phylogenetic relationships of *Schizothorax* and the species of *Schizothorax* contained in the phylogenetic analyses were incomplete. In present study, the results supported that the *S. kozlovi* was closely related to *S.chongi*, which is consistent with the study by Rustam et al. [56]. Moreover, the topologies of phylogenetic tree divided the *Schizothorax* into four clades and did not support classification based on morphology which suggested that the *Schizothorax* can be divided into two subgenera of *Schizothorax* and *Racoma*, or into three groups. This result is similar to He and Chen’s [25] analyses of the phylogenetic relationships of *Schizothorax* based on a single mark of Cyt b. He and Chen [25] and Briolay et al. [69] also reported that adaptive evolution and interbreeding of closely related species can also have impacts on inferring phylogenetic relationships in *Schizothorax*. The results reminded us that it is easy to be biased in the classification of *Schizothorax* based solely on morphological or phylogenetic methods, and it is also not sufficiently accurate to infer phylogenetic relationships from a single or a few marks. Therefore, more samples and more sequence data are required to reconstruct a more reliable phylogenetic framework and should be combined with morphological features to accurately determine the status and relationships among species of *Schizothorax*.

## 5. Conclusions

The present study reported the complete mitochondrial genome of *S. kozlovi* for the first time. The mitochondrial genome had a total size of 16,585 bp and contained thirteen PCGs, twenty-two tRNAs, two rRNAs, and two non-coding regions for OL and CR. The *Schizothorax* could be classified into four clades according to phylogenetic tree, which did not support the classification which divided *Schizothorax* into two subgenera or three groups based on morphology. In addition, the topologies of the phylogenetic tree showed that the *S. kozlovi* and *S. chongi* formed a sister group. The results of the present study have provided basic data and references for the management and conservation of *S. kozlovi* and *Schizothorax*.

## Figures and Tables

**Table 1:** The list of species included in this study for phylogenetic analyses.

Species	Size (bp)	Accession Number
*Schizothorax argentatus*	16,587	NC_061395.1
*Schizothorax biddulphi*	16,588	OR812523.1
*Schizothorax chongi*	16,584	NC_024621.1
*Schizothorax curvilabiatus*	16,578	NC_035994.1
*Schizothorax davidi*	16,576	NC_026205.1
*Schizothorax dolichonema*	16,583	NC_023979.1
*Schizothorax esocinus*	16,591	KT210882.1
*Schizothorax eurystomus*	16,590	ON920824.1
*Schizothorax gongshanensis*	16,591	NC_031803.1
*Schizothorax grahami*	16,584	NC_029708.1
*Schizothorax griseus*	16,586	NC_046462.1
*Schizothorax gulinensis*	16,587	NC_079827.1
*Schizothorax integrilabiatus*	16,588	NC_036746.1
*Schizothorax kozlovi*	16,585	OR416862.1
*Schizothorax labiatus*	16,590	KT944287.1
*Schizothorax lantsangensis*	16,580	NC_026294.1
*Schizothorax lissolabiata*	16,583	NC_027162.1
*Schizothorax macropogon*	16,588	NC_020339.1
*Schizothorax molesworthi*	16,585	MG171194.1
*Schizothorax nepalensis*	16,589	AP011207.1
*Schizothorax niger*	16,585	NC_022866.1
*Schizothorax nukiangensis*	16,585	NC_027940.1
*Schizothorax oconnori*	16,616	KT833107.1
*Schizothorax plagiostomus*	16,576	NC_023531.1
*Schizothorax prenanti*	16,587	NC_023829.1
*Schizothorax progastus*	16,575	NC_023366.1
*Schizothorax pseudoaksaiensis*	16,586	KM243919.1
*Schizothorax richardsonii*	16,592	NC_021448.1
*Schizothorax sinensis*	16,571	NC_056907.1
*Schizothorax taliensis*	16,578	NC_037516.1
*Schizothorax waltoni*	16,589	NC_020606.1
*Schizothorax wangchiachii*	16,593	NC_020360.1
*Schizothorax yunnanensis paoshanensis*	16,585	KP892531.1
*Spinibarbus denticulatus*	16,589	AP013335.1
*Spinibarbus sinensis*	16,591	NC_022465.1

**Table 2:** The organization of the complete mitochondrial genome of *S. kozlovi*. The thirteen PCGs (nad1, nad2, cox1, cox2, atp8, atp6, cox3, nad3, nad4l, nad4, nad5, nad6, cob), two rRNAs (rrnS, rrnL), twenty-two tRNAs (trnF, trnV, trnL2, trnI, trnQ, trnM, trnW, trnA, trnN, trnC, trnY, trnS2, trnD, trnK, trnG, trnR, trnH, trnS1, trnL1, trnE, trnT, trnP), and two non-coding regions of OL and CR are listed in the rows with standard abbreviations, respectively.

Gene	Location	Size (bp)	Intergenic Nucleotide	Start Codon	Stop Codon	Anticodon	Strand
trnF	1–69	69	0	---	---	GAA	H
rrnS	70–1023	954	2	---	---	---	H
trnV	1026–1097	72	22	---	---	TAC	H
rrnL	1120–2748	1629	24	---	---	---	H
trnL2	2773–2848	76	0	---	---	TAA	H
nad1	2849–3823	975	4	ATG	TAA	---	H
trnI	3828–3899	72	−2	---	---	GAT	H
trnQ	3898–3968	71	2	---	---	TTG	L
trnM	3971–4039	69	0	---	---	CAT	H
nad2	4040–5086	1047	−2	ATG	TAG	---	H
trnW	5085–5155	71	2	---	---	TCA	H
trnA	5158–5226	69	1	---	---	TGC	L
trnN	5228–5300	73	2	---	---	GTT	L
OL	5303–5334	32	−1	---	---	---	H
trnC	5334–5400	67	−1	---	---	GCA	L
trnY	5400–5470	71	1	---	---	GTA	L
cox1	5472–7022	1551	0	GTG	TAA	---	H
trnS2	7023–7093	71	3	---	---	TGA	L
trnD	7097–7168	72	12	---	---	GTC	H
cox2	7181–7871	691	0	ATG	T--	---	H
trnK	7872–7947	76	1	---	---	TTT	H
atp8	7949–8113	165	−7	ATG	TAG	---	H
atp6	8107–8790	684	−1	ATG	TAA	---	H
cox3	8790–9575	786	−1	ATG	TAA	---	H
trnG	9575–9646	72	0	---	---	TCC	H
nad3	9647–9997	351	−2	ATG	TAG	---	H
trnR	9996–10,065	70	0	---	---	TCG	H
nad4l	10,066–10,362	297	−7	ATG	TAA	---	H
nad4	10,356–11,736	1381	0	ATG	T--	---	H
trnH	11,737–11,805	69	0	---	---	GTG	H
trnS1	11,806–11,873	68	1	---	---	GCT	H
trnL1	11,875–11,947	73	3	---	---	TAG	H
nad5	11,951–13,774	1824	−4	ATG	TAA	---	H
nad6	13,771–14,292	522	0	ATG	TAA	---	L
trnE	14,293–14,361	69	4	---	---	TTC	L
cob	14,366–15,506	1141	0	ATG	T--	---	H
trnT	15,507–15,578	72	−1	---	---	TGT	H
trnP	15,578–15,647	70	15	---	---	TGG	L
CR	15,663–16,483	821	102	---	---	---	H

Note: “H” represents the heavy strand; “L” represents the light strand.

**Table 3:** The nucleotide composition of the complete mitochondrial genome of *S. kozlovi*. The nucleotide composition of thirteen PCGs, two rRNAs, twenty-two tRNAs, two non-coding regions of OL and CR, and whole genome are listed in the rows.

Regions	Size (bp)	A (%)	T (%)	G (%)	C (%)	A + T (%)	AT-Skew	GC-Skew
cox1	1551	26.24	29.08	18.63	26.05	55.32	−0.05	−0.17
cox2	691	29.96	27.21	16.93	25.90	57.17	0.05	−0.21
atp8	165	33.33	29.70	12.73	24.24	63.03	0.06	−0.31
atp6	684	29.24	28.80	15.35	26.61	58.04	0.01	−0.27
cox3	786	27.61	26.84	17.56	27.99	54.45	0.01	−0.23
nad3	351	27.92	28.77	15.38	27.92	56.69	−0.02	−0.29
nad1	975	24.10	25.13	20.41	30.36	49.23	−0.02	−0.20
nad5	1824	29.22	25.16	16.01	29.61	54.38	0.07	−0.30
nad4	1381	28.39	26.36	16.58	28.67	54.75	0.04	−0.27
nad4l	297	24.92	28.62	16.84	29.63	53.54	−0.07	−0.28
nad6	522	13.22	38.12	32.18	16.48	51.34	−0.49	0.32
cob	1141	26.47	28.66	17.09	27.78	55.13	−0.04	−0.24
nad2	1047	28.37	22.25	17.57	31.81	50.62	0.12	−0.29
PCGs	11,415	27.03	27.24	17.88	27.85	54.27	−0.003	−0.22
tRNAs	1562	28.10	26.70	23.75	21.45	54.80	0.03	0.05
rRNAs	2583	34.34	20.36	21.25	24.04	54.70	0.26	−0.06
OL	32	15.63	18.75	31.25	34.38	34.38	−0.09	−0.05
CR	821	33.13	33.74	14.01	19.12	66.87	−0.01	−0.15
genome	16,585	29.59	25.42	17.94	27.05	55.01	0.08	−0.20

## Data Availability

Data have been submitted to the GenBank database with accession number OR416862.1 and will be available upon request.
